# 1-(2,4,6-Triisopropyl­phen­yl)ethanone

**DOI:** 10.1107/S1600536811038293

**Published:** 2011-09-30

**Authors:** Amber D. Blair, Arthur D. Hendsbee, Jason D. Masuda

**Affiliations:** a The Maritimes Centre for Green Chemistry, Department of Chemistry, Saint Mary’s University, 923 Robie Street, Halifax, Nova Scotia, Canada B3H 3C3

## Abstract

The title compound, C_17_H_26_O, is a di-*ortho*-alkyl substituted phenyl ethanone that exhibits a significant twisting of the ketone fragment relative to the aromatic ring [O—C—C—C torsion angle = 89.32 (17)°] due to steric pressure from the *ortho*-isopropyl groups. One *ortho-* and the *para*-isopropyl group exhibit orientational disorder with a refined site occupancy factor of 0.562 (3):0.438 (3).

## Related literature

There are two examples in the literature of crystallo­graph­ically characterized *ortho*-substituted phenyl ethano­nes, see: van Koningsveld *et al.* (1987 ▶); Padmanabhan *et al.* (1986 ▶); De Ridder & Schenk (1995 ▶). For the preparation, see: Delair *et al.* (1996 ▶). For the use of the title mol­ecule in the preparation of 2-ethynyl-1,3,5-triisopropyl­benzene, see: Tani *et al.* (1963 ▶). For some related ligands containing *ortho*-isopropyl groups, see: Boeré & Masuda (2002 ▶); Boeré *et al.* (2008 ▶); Giffin *et al.* (2010*a* ▶,*b* ▶, 2011 ▶).
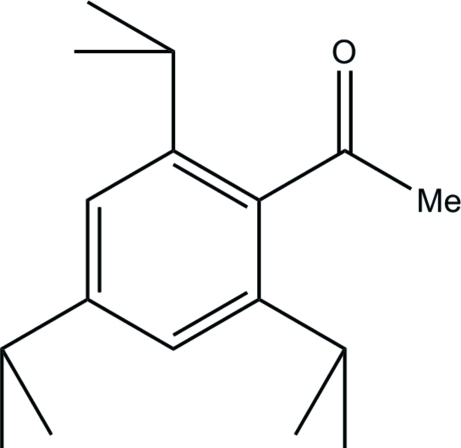


## Experimental

### 

#### Crystal data

C_17_H_26_O
*M* *_r_* = 246.38
Monoclinic, 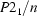

*a* = 5.8590 (12) Å
*b* = 20.248 (4) Å
*c* = 13.148 (3) Å
β = 92.568 (2)°
*V* = 1558.2 (6) Å^3^
*Z* = 4
Mo *K*α radiation
μ = 0.06 mm^−1^
*T* = 129 K
0.45 × 0.41 × 0.35 mm

#### Data collection

Bruker APEXII CCD diffractometer
Absorption correction: multi-scan (*SADABS*; Bruker, 2008 ▶) *T*_min_ = 0.972, *T*_max_ = 0.978
10949 measured reflections
3047 independent reflections
2460 reflections with *I* > 2σ(*I*)
*R*_int_ = 0.030

#### Refinement

*R*[*F* ^2^ > 2σ(*F* ^2^)] = 0.048
*wR*(*F* ^2^) = 0.120
*S* = 1.05
3047 reflections
214 parameters
21 restraints
H-atom parameters constrained
Δρ_max_ = 0.19 e Å^−3^
Δρ_min_ = −0.27 e Å^−3^

### 

Data collection: *APEX2* (Bruker, 2008 ▶); cell refinement: *SAINT* (Bruker, 2008 ▶); data reduction: *SAINT*; program(s) used to solve structure: *SHELXS97* (Sheldrick, 2008 ▶); program(s) used to refine structure: *SHELXL97* (Sheldrick, 2008 ▶); molecular graphics: *ORTEP-3 for Windows* (Farrugia, 1997 ▶) and *Mercury* (Macrae *et al.*, 2008 ▶); software used to prepare material for publication: *SHELXTL* (Bruker, 2008 ▶) and *publCIF* (Westrip, 2010 ▶).

## Supplementary Material

Crystal structure: contains datablock(s) I, global. DOI: 10.1107/S1600536811038293/nr2011sup1.cif

Structure factors: contains datablock(s) I. DOI: 10.1107/S1600536811038293/nr2011Isup2.hkl

Supplementary material file. DOI: 10.1107/S1600536811038293/nr2011Isup3.mol

Supplementary material file. DOI: 10.1107/S1600536811038293/nr2011Isup4.cml

Additional supplementary materials:  crystallographic information; 3D view; checkCIF report

## Figures and Tables

**Fig. 1.**
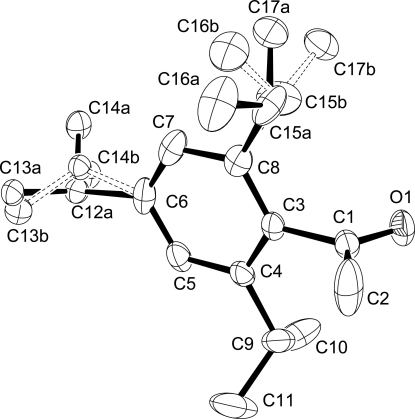
The molecular structure of 1-(2,4,6-triisopropylphenyl)ethanone, with atom labels and 50% probability displacement ellipsoids for non-H atoms. Hydrogen atoms have been removed for clarity.

**Fig. 2.**
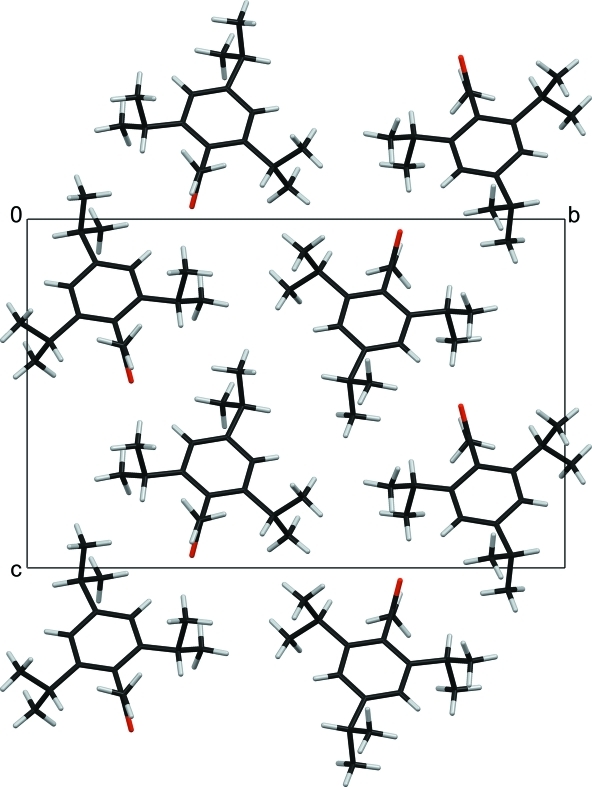
A packing diagram of 1-(2,4,6-triisopropylphenyl)ethanone viewed down the a axis. The minor disordered isopropyl groups are omitted for clarity.

## supplementary crystallographic information

### Comment

We have had a long standing interest in the impact of sterically bulky *ortho*-alkyl groups on the conformation of ligands containing aryl rings, and in particular, those with *ortho*-isopropyl substituents (Boeré & Masuda, 2002; Boeré *et al.*, 2008; Giffin *et al.*, 2010*a*; Giffin, *et al.*, 2010*b*; Giffin *et al.*, 2011). The steric impact of these *ortho*-isopropyl groups has proven important in the stabilization of many reactive functional groups. In our continuing interest in these systems we have prepared and studied the title compound which is an intermediate to the related acetylene, 2-ethynyl-1,3,5-triisopropylbenzene (Tani *et al.*, 1963).

There are few solid state structures of *ortho*-alkyl substituted ethanones reported in the literature. One example is 1-*tert*-Butyl-4-acetyl-3,5-dimethyl-2,6-dinitrobenzene that exhibit O—C—C_Ar_—C_Ar_ angles in the range of 77.50° to 84.12°(Padmanabhan *et al.*, 1986; De Ridder & Schenk, 1995). Another example is 4-*tert*-Butyl-2,6-dimethylacetophenone that has a O—C—C_Ar_—C_Ar_ angle of 79.58° (van Koningsveld *et al.*, 1987). The title compound the ketone fragment is nearly perpendicular to the aryl ring with a torsion angle of 89.32 (17)° between the ketone C=O and the aryl ring (O1—C1—C3—C4). This can be attributed to the increased steric pressure of the *ortho*-isopropyl groups relative to the *ortho*-methyl groups in the previous two examples.

One *ortho*- and the *para*-isopropyl group exhibit two-site disorder with a refined site occupancy factor of 0.562 (3):0.438 (3). A careful look at the packing of the molecule reveals no typical hydrogen bonding and lacks significant intermolecular interactions (Figure 2).

### Experimental

The title compound was prepared following literature methods (Delair *et al*, 1996) and was crystallized as large needles from a hot methanol solution cooled to room temperature.

### Refinement

The hydrogen atoms were placed in geometrically idealized positions of 0.98Å (methyl C—H), 0.95Å (aromatic C—H) and 1.00Å (methine C—H) and constrained to ride on the parent atom with *U*_iso_(H) = 1.2 *U*_eq_(C) for aromatic and tertiary protons as well as *U*_iso_(H) = 1.5 *U*_eq_(C) for the methyl groups. Two of the three isopropyl groups were modeled with two-site disorder. The isopropyl groups containing atoms C12A, C13A, C14A and C12B, C13B, C14B as well as C15A, C16A, C17A and C15B, C16B, C17B were modeled with a refined site occupancy factor of 0.562 (3):0.438 (3). In order to obtain satisfactory thermal parameters for the disordered part of the molecule DELU commands were used on the atoms C12A, C12B, C13A, C13B, C14A, C14B and C15A, C15B, C16A, C16B, C17A, C17B respectively for each isopropyl group. Unit-cell parameters were determinedd using a θ range of 2.54 to 27.83° however, in order to obtian a reasonable level of data completeness, θ was limited to 26.00° for refinement.

### Crystal data

**Table:**

C_17_H_26_O	*F*(000) = 544
*M**_r_* = 246.38	*D*_x_ = 1.051 Mg m^−^^3^
Monoclinic, *P*2_1_/*n*	Mo *K*α radiation, λ = 0.71073 Å
Hall symbol: -P 2yn	Cell parameters from 3864 reflections
*a* = 5.8590 (12) Å	θ = 2.5–27.8°
*b* = 20.248 (4) Å	µ = 0.06 mm^−^^1^
*c* = 13.148 (3) Å	*T* = 129 K
β = 92.568 (2)°	Block, colourless
*V* = 1558.2 (6) Å^3^	0.45 × 0.41 × 0.35 mm
*Z* = 4	

### Data collection

**Table:**

Bruker APEXII CCD diffractometer	3047 independent reflections
Radiation source: fine-focus sealed tube	2460 reflections with *I* > 2σ(*I*)
graphite	*R*_int_ = 0.030
φ and ω scans	θ_max_ = 26.0°, θ_min_ = 2.0°
Absorption correction: multi-scan (*SADABS*; Bruker, 2008)	*h* = −7→7
*T*_min_ = 0.972, *T*_max_ = 0.978	*k* = −24→24
10949 measured reflections	*l* = −16→13

### Refinement

**Table:**

Refinement on *F*^2^	Primary atom site location: structure-invariant direct methods
Least-squares matrix: full	Secondary atom site location: difference Fourier map
*R*[*F*^2^ > 2σ(*F*^2^)] = 0.048	Hydrogen site location: inferred from neighbouring sites
*wR*(*F*^2^) = 0.120	H-atom parameters constrained
*S* = 1.05	*w* = 1/[σ^2^(*F*_o_^2^) + (0.0417*P*)^2^ + 0.6687*P*] where *P* = (*F*_o_^2^ + 2*F*_c_^2^)/3
3047 reflections	(Δ/σ)_max_ < 0.001
214 parameters	Δρ_max_ = 0.19 e Å^−^^3^
21 restraints	Δρ_min_ = −0.27 e Å^−^^3^

### Special details

**Table:**

Geometry. All e.s.d.'s (except the e.s.d. in the dihedral angle between two l.s. planes) are estimated using the full covariance matrix. The cell e.s.d.'s are taken into account individually in the estimation of e.s.d.'s in distances, angles and torsion angles; correlations between e.s.d.'s in cell parameters are only used when they are defined by crystal symmetry. An approximate (isotropic) treatment of cell e.s.d.'s is used for estimating e.s.d.'s involving l.s. planes.
Refinement. Refinement of *F*^2^ against ALL reflections. The weighted *R*-factor *wR* and goodness of fit *S* are based on *F*^2^, conventional *R*-factors *R* are based on *F*, with *F* set to zero for negative *F*^2^. The threshold expression of *F*^2^ > σ(*F*^2^) is used only for calculating *R*-factors(gt) *etc*. and is not relevant to the choice of reflections for refinement. *R*-factors based on *F*^2^ are statistically about twice as large as those based on *F*, and *R*- factors based on ALL data will be even larger.

### Fractional atomic coordinates and isotropic or equivalent isotropic displacement parameters (Å^2^)

**Table:**

	*x*	*y*	*z*	*U*_iso_*/*U*_eq_	Occ. (<1)
O1	0.01390 (17)	0.19352 (5)	0.46376 (7)	0.0360 (3)	
C1	−0.0742 (2)	0.17979 (7)	0.38182 (10)	0.0274 (3)	
C2	−0.3278 (3)	0.17911 (13)	0.36357 (14)	0.0645 (7)	
H2A	−0.4011	0.1937	0.4253	0.097*	
H2B	−0.3783	0.1342	0.3462	0.097*	
H2C	−0.3705	0.2090	0.3073	0.097*	
C3	0.0664 (2)	0.16216 (7)	0.29222 (10)	0.0250 (3)	
C4	0.1265 (2)	0.09627 (7)	0.27715 (11)	0.0298 (3)	
C5	0.2545 (2)	0.08078 (8)	0.19350 (11)	0.0337 (4)	
H5A	0.2963	0.0361	0.1827	0.040*	
C6	0.3226 (2)	0.12825 (9)	0.12577 (11)	0.0340 (4)	
C7	0.2595 (2)	0.19336 (8)	0.14225 (11)	0.0345 (4)	
H7A	0.3045	0.2265	0.0960	0.041*	
C8	0.1318 (2)	0.21148 (7)	0.22491 (10)	0.0284 (3)	
C9	0.0604 (3)	0.04223 (8)	0.35037 (14)	0.0458 (5)	
H9A	−0.0378	0.0627	0.4021	0.055*	
C10	0.2720 (4)	0.01420 (11)	0.40679 (19)	0.0743 (7)	
H10A	0.3480	0.0492	0.4471	0.111*	
H10B	0.3773	−0.0032	0.3574	0.111*	
H10C	0.2268	−0.0215	0.4521	0.111*	
C11	−0.0781 (4)	−0.01245 (10)	0.2970 (2)	0.0781 (8)	
H11A	−0.2156	0.0065	0.2634	0.117*	
H11B	−0.1222	−0.0453	0.3473	0.117*	
H11C	0.0146	−0.0337	0.2461	0.117*	
C12A	0.4554 (7)	0.09901 (19)	0.0358 (4)	0.0262 (8)	0.562 (3)
H12A	0.4883	0.0512	0.0487	0.031*	0.562 (3)
C13A	0.3133 (6)	0.1066 (2)	−0.0634 (3)	0.0268 (7)	0.562 (3)
H13A	0.4034	0.0925	−0.1205	0.040*	0.562 (3)
H13B	0.2693	0.1530	−0.0726	0.040*	0.562 (3)
H13C	0.1756	0.0793	−0.0609	0.040*	0.562 (3)
C14A	0.6817 (4)	0.13736 (14)	0.0294 (2)	0.0299 (7)	0.562 (3)
H14A	0.7695	0.1193	−0.0259	0.045*	0.562 (3)
H14B	0.7706	0.1331	0.0940	0.045*	0.562 (3)
H14C	0.6488	0.1841	0.0160	0.045*	0.562 (3)
C12B	0.4652 (9)	0.1237 (2)	0.0327 (5)	0.0238 (10)	0.438 (3)
H12B	0.5066	0.1689	0.0090	0.029*	0.438 (3)
C14B	0.6776 (5)	0.08476 (18)	0.0610 (3)	0.0307 (9)	0.438 (3)
H14D	0.7730	0.1098	0.1105	0.046*	0.438 (3)
H14E	0.7633	0.0766	−0.0001	0.046*	0.438 (3)
H14F	0.6347	0.0425	0.0910	0.046*	0.438 (3)
C13B	0.3143 (10)	0.0881 (3)	−0.0497 (4)	0.0287 (14)*	0.438 (3)
H13D	0.4046	0.0791	−0.1090	0.043*	0.438 (3)
H13E	0.1841	0.1163	−0.0700	0.043*	0.438 (3)
H13F	0.2584	0.0465	−0.0222	0.043*	0.438 (3)
C15A	0.0614 (13)	0.2804 (4)	0.2368 (7)	0.0403 (10)	0.562 (3)
H15A	−0.0094	0.2858	0.3041	0.048*	0.562 (3)
C16A	−0.1203 (7)	0.2960 (2)	0.1501 (3)	0.0578 (10)	0.562 (3)
H16A	−0.1752	0.3414	0.1576	0.087*	0.562 (3)
H16B	−0.2488	0.2653	0.1541	0.087*	0.562 (3)
H16C	−0.0511	0.2912	0.0840	0.087*	0.562 (3)
C17A	0.2662 (6)	0.32461 (14)	0.2330 (3)	0.0495 (9)	0.562 (3)
H17A	0.3818	0.3106	0.2847	0.074*	0.562 (3)
H17B	0.2208	0.3703	0.2462	0.074*	0.562 (3)
H17C	0.3298	0.3218	0.1654	0.074*	0.562 (3)
C15B	0.0787 (18)	0.2866 (5)	0.2488 (10)	0.044 (2)	0.438 (3)
H15B	−0.0508	0.2864	0.2958	0.053*	0.438 (3)
C16B	0.0019 (8)	0.3298 (2)	0.1582 (3)	0.0445 (10)	0.438 (3)
H16D	−0.1275	0.3088	0.1211	0.067*	0.438 (3)
H16E	0.1286	0.3350	0.1126	0.067*	0.438 (3)
H16F	−0.0445	0.3732	0.1827	0.067*	0.438 (3)
C17B	0.2869 (6)	0.32093 (18)	0.3094 (4)	0.0402 (10)	0.438 (3)
H17D	0.3228	0.2964	0.3723	0.060*	0.438 (3)
H17E	0.2459	0.3665	0.3260	0.060*	0.438 (3)
H17F	0.4206	0.3211	0.2671	0.060*	0.438 (3)

### Atomic displacement parameters (Å^2^)

**Table:**

	*U*^11^	*U*^22^	*U*^33^	*U*^12^	*U*^13^	*U*^23^
O1	0.0370 (6)	0.0477 (7)	0.0232 (5)	−0.0009 (5)	−0.0008 (4)	−0.0086 (5)
C1	0.0263 (7)	0.0320 (7)	0.0240 (7)	−0.0024 (6)	0.0019 (5)	−0.0061 (6)
C2	0.0251 (8)	0.131 (2)	0.0382 (10)	−0.0011 (10)	0.0074 (7)	−0.0324 (11)
C3	0.0169 (6)	0.0364 (8)	0.0215 (7)	−0.0047 (5)	−0.0007 (5)	−0.0064 (6)
C4	0.0251 (7)	0.0345 (8)	0.0302 (8)	−0.0103 (6)	0.0061 (6)	−0.0112 (6)
C5	0.0268 (7)	0.0419 (8)	0.0330 (8)	−0.0057 (6)	0.0067 (6)	−0.0162 (7)
C6	0.0176 (6)	0.0633 (10)	0.0212 (7)	−0.0002 (6)	0.0001 (5)	−0.0076 (7)
C7	0.0210 (7)	0.0589 (10)	0.0235 (7)	0.0008 (6)	−0.0013 (5)	0.0088 (7)
C8	0.0197 (6)	0.0397 (7)	0.0253 (7)	0.0006 (5)	−0.0048 (5)	0.0021 (6)
C9	0.0527 (10)	0.0298 (8)	0.0574 (11)	−0.0150 (7)	0.0299 (9)	−0.0112 (7)
C10	0.0848 (16)	0.0629 (14)	0.0770 (16)	−0.0128 (12)	0.0222 (13)	0.0352 (12)
C11	0.0690 (14)	0.0466 (11)	0.123 (2)	−0.0340 (10)	0.0532 (14)	−0.0408 (12)
C12A	0.0276 (14)	0.027 (2)	0.0246 (13)	−0.0014 (14)	0.0059 (10)	−0.002 (2)
C13A	0.0273 (14)	0.032 (2)	0.0215 (12)	−0.0052 (15)	0.0069 (10)	−0.0046 (15)
C14A	0.0219 (11)	0.0399 (17)	0.0283 (13)	0.0005 (10)	0.0048 (9)	−0.0030 (12)
C12B	0.0224 (18)	0.027 (3)	0.0221 (16)	−0.0016 (17)	0.0031 (11)	−0.003 (3)
C14B	0.0245 (15)	0.038 (2)	0.0303 (18)	0.0031 (12)	0.0035 (12)	−0.0010 (14)
C15A	0.039 (3)	0.0478 (17)	0.035 (2)	0.0202 (17)	0.0160 (19)	0.017 (2)
C16A	0.059 (2)	0.072 (3)	0.0433 (19)	0.0391 (18)	0.0063 (15)	0.0148 (18)
C17A	0.077 (2)	0.0236 (12)	0.049 (2)	0.0030 (13)	0.0153 (17)	−0.0030 (14)
C15B	0.036 (4)	0.0340 (19)	0.061 (5)	0.006 (2)	−0.013 (3)	0.011 (2)
C16B	0.049 (2)	0.0377 (19)	0.047 (2)	0.0158 (19)	0.0026 (18)	0.0073 (17)
C17B	0.044 (2)	0.0297 (17)	0.047 (3)	−0.0006 (14)	0.0028 (16)	−0.0076 (16)

### Geometric parameters (Å, °)

**Table:**

O1—C1	1.2060 (17)	C13A—H13B	0.9800
C1—C2	1.495 (2)	C13A—H13C	0.9800
C1—C3	1.5102 (19)	C14A—H14A	0.9800
C2—H2A	0.9800	C14A—H14B	0.9800
C2—H2B	0.9800	C14A—H14C	0.9800
C2—H2C	0.9800	C12B—C14B	1.506 (6)
C3—C4	1.396 (2)	C12B—C13B	1.546 (8)
C3—C8	1.399 (2)	C12B—H12B	1.0000
C4—C5	1.3942 (19)	C14B—H14D	0.9800
C4—C9	1.519 (2)	C14B—H14E	0.9800
C5—C6	1.381 (2)	C14B—H14F	0.9800
C5—H5A	0.9500	C13B—H13D	0.9800
C6—C7	1.389 (2)	C13B—H13E	0.9800
C6—C12B	1.515 (7)	C13B—H13F	0.9800
C6—C12A	1.561 (5)	C15A—C17A	1.500 (8)
C7—C8	1.396 (2)	C15A—C16A	1.557 (8)
C7—H7A	0.9500	C15A—H15A	1.0000
C8—C15A	1.466 (8)	C16A—H16A	0.9800
C8—C15B	1.587 (11)	C16A—H16B	0.9800
C9—C11	1.525 (2)	C16A—H16C	0.9800
C9—C10	1.526 (3)	C17A—H17A	0.9800
C9—H9A	1.0000	C17A—H17B	0.9800
C10—H10A	0.9800	C17A—H17C	0.9800
C10—H10B	0.9800	C15B—C16B	1.528 (11)
C10—H10C	0.9800	C15B—C17B	1.587 (10)
C11—H11A	0.9800	C15B—H15B	1.0000
C11—H11B	0.9800	C16B—H16D	0.9800
C11—H11C	0.9800	C16B—H16E	0.9800
C12A—C13A	1.524 (7)	C16B—H16F	0.9800
C12A—C14A	1.542 (5)	C17B—H17D	0.9800
C12A—H12A	1.0000	C17B—H17E	0.9800
C13A—H13A	0.9800	C17B—H17F	0.9800
			
O1—C1—C2	121.88 (13)	C12A—C14A—H14A	109.5
O1—C1—C3	121.65 (12)	C12A—C14A—H14B	109.5
C2—C1—C3	116.47 (12)	H14A—C14A—H14B	109.5
C1—C2—H2A	109.5	C12A—C14A—H14C	109.5
C1—C2—H2B	109.5	H14A—C14A—H14C	109.5
H2A—C2—H2B	109.5	H14B—C14A—H14C	109.5
C1—C2—H2C	109.5	C6—C12B—C14B	108.1 (4)
H2A—C2—H2C	109.5	C6—C12B—C13B	106.0 (4)
H2B—C2—H2C	109.5	C14B—C12B—C13B	111.7 (4)
C4—C3—C8	120.95 (13)	C6—C12B—H12B	110.3
C4—C3—C1	119.11 (12)	C14B—C12B—H12B	110.3
C8—C3—C1	119.94 (13)	C13B—C12B—H12B	110.3
C5—C4—C3	118.35 (14)	C12B—C14B—H14D	109.5
C5—C4—C9	119.96 (14)	C12B—C14B—H14E	109.5
C3—C4—C9	121.68 (13)	H14D—C14B—H14E	109.5
C6—C5—C4	122.21 (14)	C12B—C14B—H14F	109.5
C6—C5—H5A	118.9	H14D—C14B—H14F	109.5
C4—C5—H5A	118.9	H14E—C14B—H14F	109.5
C5—C6—C7	118.26 (13)	C12B—C13B—H13D	109.5
C5—C6—C12B	131.6 (2)	C12B—C13B—H13E	109.5
C7—C6—C12B	110.1 (2)	H13D—C13B—H13E	109.5
C5—C6—C12A	113.13 (19)	C12B—C13B—H13F	109.5
C7—C6—C12A	128.56 (19)	H13D—C13B—H13F	109.5
C6—C7—C8	121.78 (14)	H13E—C13B—H13F	109.5
C6—C7—H7A	119.1	C8—C15A—C17A	109.6 (5)
C8—C7—H7A	119.1	C8—C15A—C16A	107.6 (5)
C7—C8—C3	118.45 (14)	C17A—C15A—C16A	112.1 (5)
C7—C8—C15A	119.8 (3)	C8—C15A—H15A	109.2
C3—C8—C15A	121.7 (3)	C17A—C15A—H15A	109.2
C7—C8—C15B	121.5 (5)	C16A—C15A—H15A	109.2
C3—C8—C15B	119.8 (5)	C15A—C16A—H16A	109.5
C4—C9—C11	112.16 (17)	C15A—C16A—H16B	109.5
C4—C9—C10	110.56 (13)	H16A—C16A—H16B	109.5
C11—C9—C10	110.95 (17)	C15A—C16A—H16C	109.5
C4—C9—H9A	107.7	H16A—C16A—H16C	109.5
C11—C9—H9A	107.7	H16B—C16A—H16C	109.5
C10—C9—H9A	107.7	C15A—C17A—H17A	109.5
C9—C10—H10A	109.5	C15A—C17A—H17B	109.5
C9—C10—H10B	109.5	H17A—C17A—H17B	109.5
H10A—C10—H10B	109.5	C15A—C17A—H17C	109.5
C9—C10—H10C	109.5	H17A—C17A—H17C	109.5
H10A—C10—H10C	109.5	H17B—C17A—H17C	109.5
H10B—C10—H10C	109.5	C16B—C15B—C17B	109.5 (7)
C9—C11—H11A	109.5	C16B—C15B—C8	116.6 (8)
C9—C11—H11B	109.5	C17B—C15B—C8	111.5 (6)
H11A—C11—H11B	109.5	C16B—C15B—H15B	106.2
C9—C11—H11C	109.5	C17B—C15B—H15B	106.2
H11A—C11—H11C	109.5	C8—C15B—H15B	106.2
H11B—C11—H11C	109.5	C15B—C16B—H16D	109.5
C13A—C12A—C14A	109.9 (4)	C15B—C16B—H16E	109.5
C13A—C12A—C6	109.8 (3)	H16D—C16B—H16E	109.5
C14A—C12A—C6	108.0 (3)	C15B—C16B—H16F	109.5
C13A—C12A—H12A	109.7	H16D—C16B—H16F	109.5
C14A—C12A—H12A	109.7	H16E—C16B—H16F	109.5
C6—C12A—H12A	109.7	C15B—C17B—H17D	109.5
C12A—C13A—H13A	109.5	C15B—C17B—H17E	109.5
C12A—C13A—H13B	109.5	H17D—C17B—H17E	109.5
H13A—C13A—H13B	109.5	C15B—C17B—H17F	109.5
C12A—C13A—H13C	109.5	H17D—C17B—H17F	109.5
H13A—C13A—H13C	109.5	H17E—C17B—H17F	109.5
H13B—C13A—H13C	109.5		
			
O1—C1—C3—C4	89.32 (17)	C5—C4—C9—C10	66.0 (2)
C2—C1—C3—C4	−90.50 (18)	C3—C4—C9—C10	−112.65 (17)
O1—C1—C3—C8	−91.58 (17)	C5—C6—C12A—C13A	112.2 (3)
C2—C1—C3—C8	88.60 (18)	C7—C6—C12A—C13A	−64.9 (4)
C8—C3—C4—C5	0.3 (2)	C12B—C6—C12A—C13A	−77.6 (11)
C1—C3—C4—C5	179.40 (12)	C5—C6—C12A—C14A	−128.0 (2)
C8—C3—C4—C9	178.99 (13)	C7—C6—C12A—C14A	55.0 (4)
C1—C3—C4—C9	−1.9 (2)	C12B—C6—C12A—C14A	42.2 (10)
C3—C4—C5—C6	−0.1 (2)	C5—C6—C12B—C14B	−48.4 (4)
C9—C4—C5—C6	−178.78 (14)	C7—C6—C12B—C14B	130.1 (3)
C4—C5—C6—C7	−0.3 (2)	C12A—C6—C12B—C14B	−60.5 (11)
C4—C5—C6—C12B	178.1 (3)	C5—C6—C12B—C13B	71.5 (5)
C4—C5—C6—C12A	−177.6 (2)	C7—C6—C12B—C13B	−110.1 (3)
C5—C6—C7—C8	0.4 (2)	C12A—C6—C12B—C13B	59.3 (10)
C12B—C6—C7—C8	−178.4 (3)	C7—C8—C15A—C17A	−52.9 (6)
C12A—C6—C7—C8	177.3 (3)	C3—C8—C15A—C17A	129.8 (4)
C6—C7—C8—C3	−0.1 (2)	C15B—C8—C15A—C17A	53 (6)
C6—C7—C8—C15A	−177.5 (4)	C7—C8—C15A—C16A	69.2 (5)
C6—C7—C8—C15B	174.6 (4)	C3—C8—C15A—C16A	−108.1 (5)
C4—C3—C8—C7	−0.21 (19)	C15B—C8—C15A—C16A	175 (7)
C1—C3—C8—C7	−179.30 (12)	C7—C8—C15B—C16B	44.8 (8)
C4—C3—C8—C15A	177.1 (4)	C3—C8—C15B—C16B	−140.5 (6)
C1—C3—C8—C15A	−1.9 (4)	C15A—C8—C15B—C16B	−33 (6)
C4—C3—C8—C15B	−175.0 (4)	C7—C8—C15B—C17B	−81.9 (8)
C1—C3—C8—C15B	5.9 (4)	C3—C8—C15B—C17B	92.8 (8)
C5—C4—C9—C11	−58.4 (2)	C15A—C8—C15B—C17B	−160 (7)
C3—C4—C9—C11	122.94 (16)		
